# Supplementary material for: Tumor-Targeted Erythrocyte Membrane Nanoparticles for Theranostics of Triple-Negative Breast Cancer
Source: Pharmaceutics. 2023 Jan 20;15(2):350. doi: 10.3390/pharmaceutics15020350 (PMC9966336; doi:10.3390/pharmaceutics15020350)
Supplement: Supplementary file 1 [file pharmaceutics-15-00350-s001.zip › pharmaceutics-2082930-supplementary.pdf]

Supporting Information for Pharmaceutics

Development of tumor-targeted nanoparticles using erythrocyte membranes for triple-negative breast cancer theranosis

Moon Jung Choi[†], *Yeon Kyung Lee*[1], *Kang Chan Choi*[1], *Do Hyun Lee*[1], *Hwa Yeon Jeong*[1], *Seong Jae Kang*[1], *Min Woo Kim*[1], *Young Myoung You*[1], *Chan Su Im*[1], *Tae Sup Lee*[2], *and Yong Serk Park*[1*]

[1]Department of Biomedical Laboratory Science, Yonsei University, Wonju, Republic of Korea.

[2]Division of RI-Convergence Research, Korea Institute of Radiological and Medical Science, Seoul, Republic of Korea.

[*]Corresponding Author: YS Park, parkys@yonsei.ac.kr

Keywords: erythrocyte-derived nanoparticle, drug delivery system, triple-negative breast cancer, tumor-targeted therapy, quantum dots

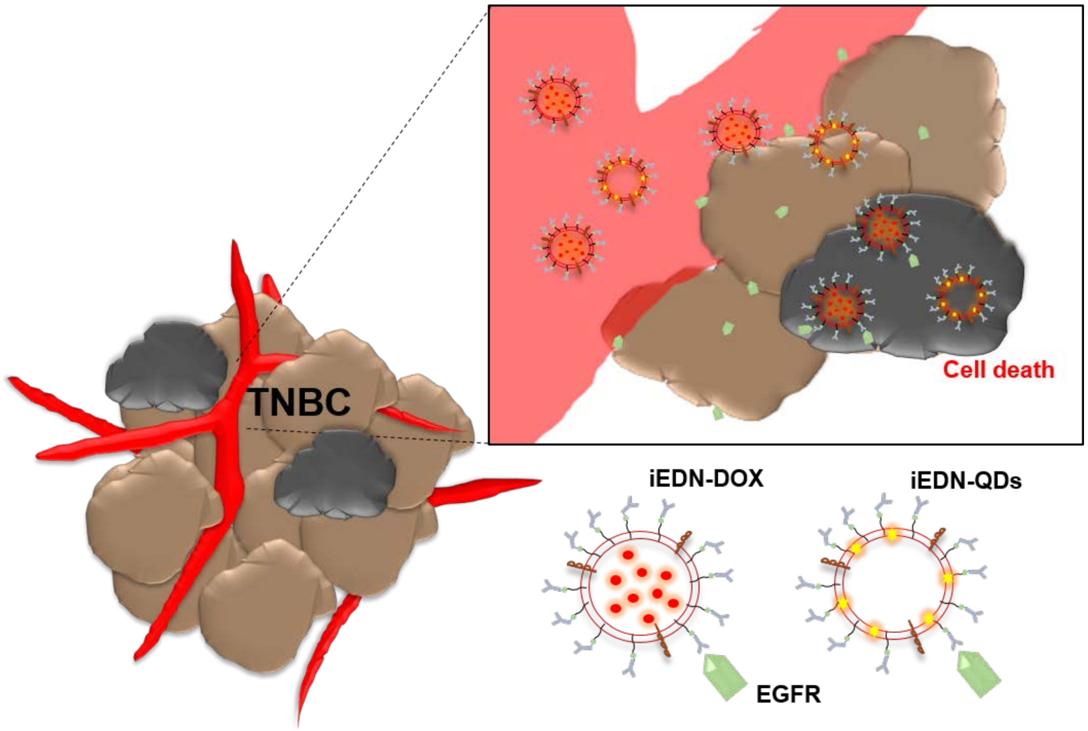

Figure S1. Schematic of the anti-cancer theranosis mechanism of tumor-targeted erythrocyte-derived nanoparticles (EDNs). Erythrocytes were treated with a hypotonic solution followed by sonication to make EDNs. The resulting erythrocyte membrane particles were conjugated with tumor-targeting antibodies (iEDN). Then, an anti-cancer drug, doxorubicin (DOX) and QDs were encapsulated into the EDNs (iEDN-DOX, iEDN-QD).

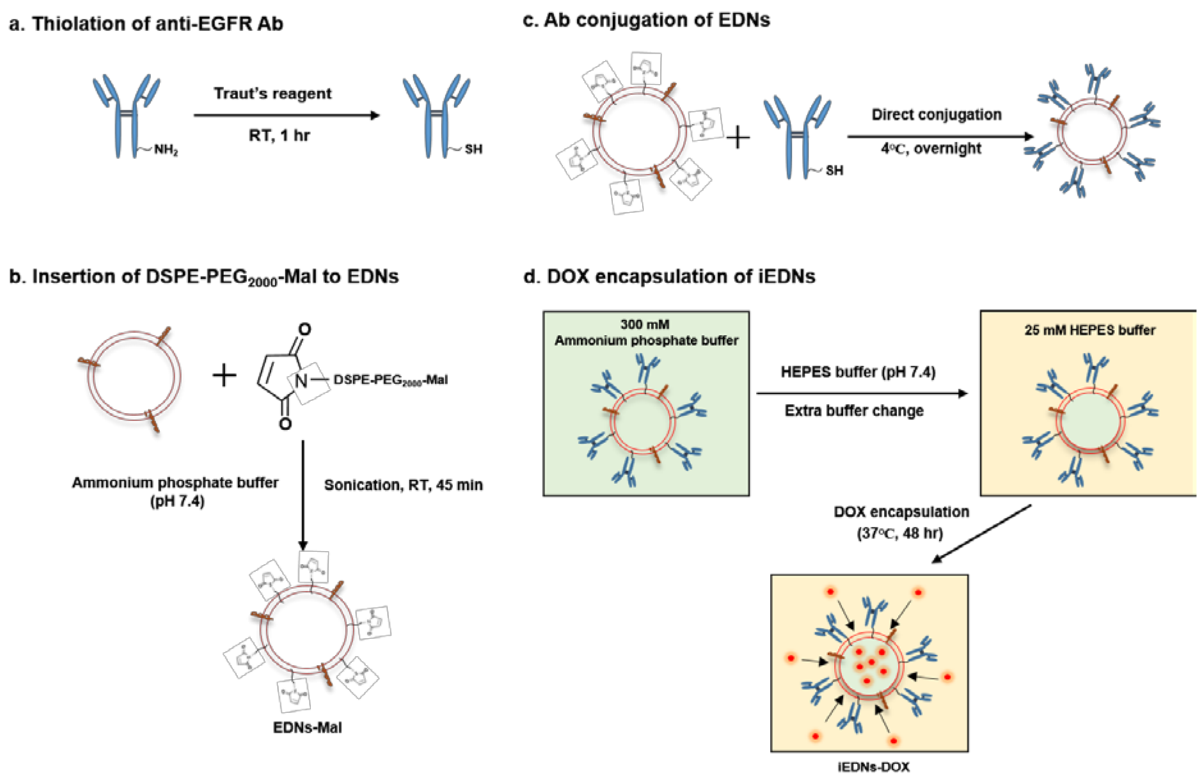

Figure S2. Schematic of the preparation process of immuno-EDNs (iEDNs)-DOX. (a) Anti-epidermal growth factor receptor (anti-EGFR) antibodies were thiolated with Traut's reagent. (b) DSPE-PEG2000-Mal was inserted into EDNs by sonication and (c) thiolated antibodies were conjugated to the maleimide moiety on the EDNs. (d) DOX was encapsulated by the phosphate gradient method. For preparation of EDNs-quantum dots (QDs), hydrophobic QDs were mixed with DSPE-mPEG2000 and added to erythrocyte ghosts before sonication.

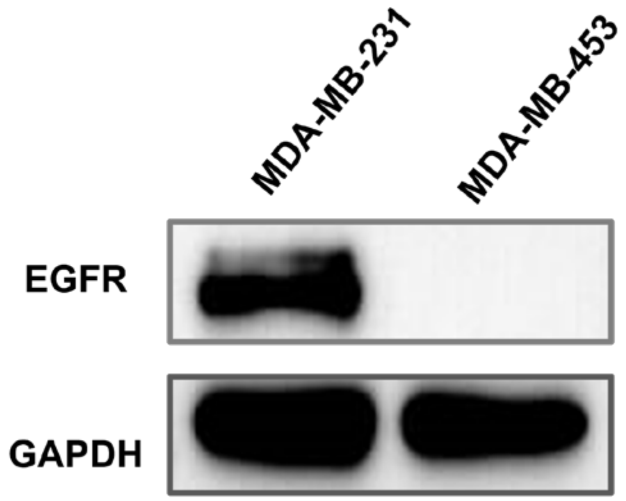

Figure S3. Expression levels of EGFRs in MDA-MB-231 and MDA-MB-453 cells. The expression levels of EGFRs in MDA-MB-231 and MDA-MB-453 cells were analyzed by western blotting. Glyceraldehyde-3-phosphate dehydrogenase (GAPDH) was used as an internal control.

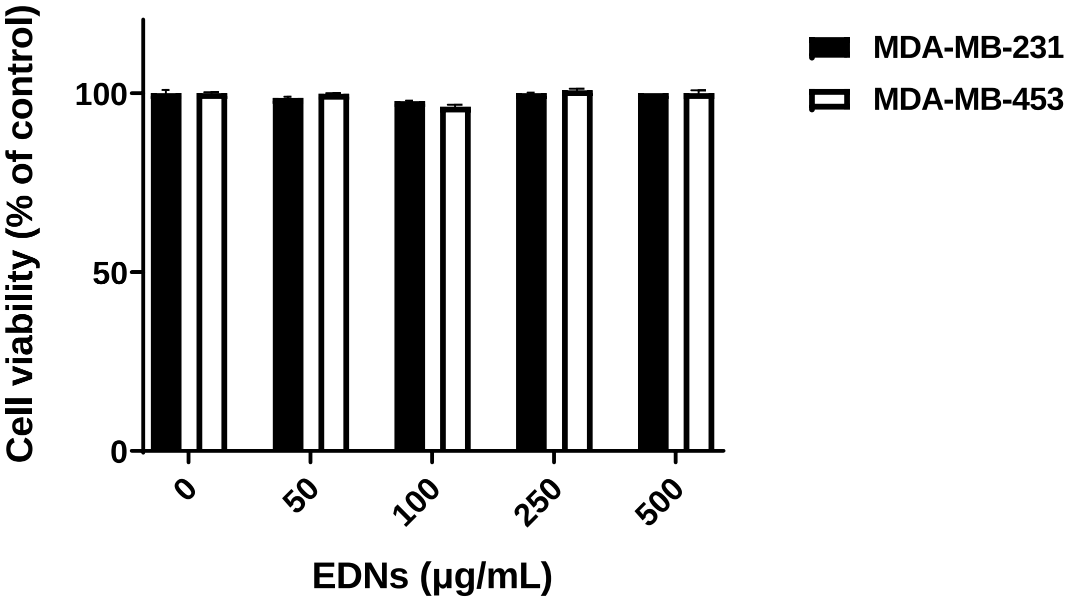

Figure S4. *In vitro* cytotoxicity of EDNs. Varied amounts of EDNs were used to treat MDA-MB-231 and MDA-MB-453 cells for 24 h, and the cell viability was analyzed by the cell counting kit-8 (CCK-8) assay. Error bars represent the mean ± standard deviation (S.D.) for three separate experiments.
